# Replication Protein A Presents Canonical Functions and Is Also Involved in the Differentiation Capacity of *Trypanosoma cruzi*

**DOI:** 10.1371/journal.pntd.0005181

**Published:** 2016-12-16

**Authors:** Raphael Souza Pavani, Marcelo Santos da Silva, Carlos Alexandre Henrique Fernandes, Flavia Souza Morini, Christiane Bezerra Araujo, Marcos Roberto de Mattos Fontes, Osvaldo Augusto Sant’Anna, Carlos Renato Machado, Maria Isabel Cano, Stenio Perdigão Fragoso, Maria Carolina Elias

**Affiliations:** 1 Laboratório Especial de Ciclo Celular, Instituto Butantan, São Paulo, São Paulo, Brazil; 2 Center of Toxins, Immune Response and Cell Signaling—CeTICS, Instituto Butantan, São Paulo, São Paulo, Brazil; 3 Departamento de Física e Biofísica, Instituto de Biociências, Universidade Estadual Paulista Júlio de Mesquita Filho -UNESP, Botucatu, São Paulo, Brazil; 4 Instituto Carlos Chagas, Fiocruz-PR, Brazil; 5 Laboratório de Imunoquímica, Instituto Butantan, São Paulo, São Paulo, Brazil; 6 Departamento de Bioquímica e Imunologia, ICB, Universidade Federal de Minas Gerais, Belo Horizonte, Minas Gerais, Brazil; 7 Departamento de Genética, Instituto de Biociências, Universidade Estadual Paulista Julio Mesquita Filho—UNESP, Botucatu, São Paulo, Brazil; Instituto de Investigaciones Biotecnológicas, ARGENTINA

## Abstract

Replication Protein A (RPA), the major single stranded DNA binding protein in eukaryotes, is composed of three subunits and is a fundamental player in DNA metabolism, participating in replication, transcription, repair, and the DNA damage response. In human pathogenic trypanosomatids, only limited studies have been performed on RPA-1 from *Leishmania*. Here, we performed *in silico*, *in vitro* and *in vivo* analysis of *Trypanosoma cruzi* RPA-1 and RPA-2 subunits. Although computational analysis suggests similarities in DNA binding and Ob-fold structures of RPA from *T*. *cruzi* compared with mammalian and fungi RPA, the predicted tridimensional structures of *T*. *cruzi* RPA-1 and RPA-2 indicated that these molecules present a more flexible tertiary structure, suggesting that *T*. *cruzi* RPA could be involved in additional responses. Here, we demonstrate experimentally that the *T*. *cruzi* RPA complex interacts with DNA via RPA-1 and is directly related to canonical functions, such as DNA replication and DNA damage response. Accordingly, a reduction of TcRPA-2 expression by generating heterozygous knockout cells impaired cell growth, slowing down S-phase progression. Moreover, heterozygous knockout cells presented a better efficiency in differentiation from epimastigote to metacyclic trypomastigote forms and metacyclic trypomastigote infection. Taken together, these findings indicate the involvement of TcRPA in the metacyclogenesis process and suggest that a delay in cell cycle progression could be linked with differentiation in *T*. *cruzi*.

## Author Summary

*Trypanosoma cruzi* is the etiological agent of Chagas disease. During its life cycle, this parasite alternates between proliferative/non-infective forms and forms that are infective but not able to proliferate. Some stressors, such as acidic pH and starvation, trigger the transition from one form to another; however, many molecules involved in this response remain to be identified. Replication Protein A (RPA) is a single stranded DNA binding protein involved in many functions of DNA metabolism, such as DNA replication and repair. Although RPA is well characterized in yeast and mammalian cells, nothing is known about this heterotrimer in *T*. *cruzi*. Here, we demonstrated that *T*. *cruzi* RPA is involved in canonical functions of DNA metabolism. Accordingly, when we reduced the expression levels of subunit 2 of TcRPA (TcRPA-2) the growth of the replicative form of *T*. *cruzi* was compromised. Moreover, we observed that the impairment of cell growth is linked with the differentiation process because the reduction of the level of TcRPA-2 increased the capacity of the proliferative epimastigote form to differentiate into an infective metacyclic trypomastigote one. In conclusion, TcRPA has canonical functions in the ancient eukaryote *T*. *cruzi* and is also involved in the control of life cycle progression.

## Introduction

*Trypanosoma cruzi* is the etiological agent of Chagas disease that infects 8 to 10 million people worldwide. Alternating between mammalian and insect hosts, the parasite faces changing environmental conditions, including thermal shifting, nutritional availability, and osmotic and oxidative stresses (for review [1]). Based on its success to establish chronic infections, one can infer that *T*. *cruzi* possesses adaptive mechanisms to respond to environmental changes. A complex life cycle most likely compensates for the variations in extracellular conditions. *T*. *cruzi* has four developmental stages, differing in shape, metabolism, replicative and infective capacity. *T*. *cruzi* epimastigotes are a non-infective life cycle stage of the parasite that proliferate by binary fission in the guts of *Triatoma infestans* insects. These epimastigotes then transform into the infective, non-proliferative metacyclic trypomastigotes forms in the insect hindgut. When the insect vector bites a mammalian host, they eliminate the infective forms in their feces. This allows the parasites to penetrate the wounded skin and enter into the mammalian host’s circulatory system. Within the bloodstream, the metacyclic trypomastigotes infect mammalian cells and transform into replicative, spherically shaped amastigotes. Amastigotes proliferate inside the infected cells until they transform into non-replicative trypomastigotes. The life cycle is completed when an insect vector bites an infected mammalian host and takes up trypomastigotes within the blood that then transform into epimastigotes inside the insect gut ([2]). Although it has been previously described that some stressors, such as acidic pH and starvation, trigger the transition from one form to another [3], the molecular bases involved in this response remain to be elucidated, such as which molecules are sensors or transducers of these differentiation pathways.

In other eukaryotes, cell cycle regulation may be a relevant mechanism in the transition from a proliferative to differentiation state of a cell. In vertebrates, inhibition of the cell cycle regulator cyclin dependent kinase (CDK) in neuroepithelial cells induces premature differentiation [4]. In the same way, inactivation of regulators of cell cycle, and the DNA metabolism-involved replication protein A (RPA) in *Drosophila*, is required for proper neuroepithelial differentiation into neuroblasts [5]. Therefore, we assessed whether impairment of cell proliferation through cell cycle alteration by RPA reduction could affect the *T*. *cruzi* differentiation from a replicative (epimastigote) to a non-replicative (metacyclic trypomastigote) stage.

RPA is the major single-stranded binding protein from eukaryotes and is a fundamental player in DNA metabolism, participating in replication, transcription, and the DNA damage response [6][7][8][9]. RPA is a conserved heterotrimeric complex composed of subunits RPA-1, RPA-2 and RPA-3. One of the major structural features of RPA is the presence of the oligonucleotide/oligosaccharide binding folds (OBF, also called DNA binding domains, DBD, in human RPA) within the subunits. This OB fold structure consists of beta sheets that form beta-barrel structures that can wrap around ssDNA [10]. In mammals and yeast, RPA-1 is the main subunit responsible for RPA-DNA interaction [11][12]. The major role of RPA-2 is to regulate RPA activity in different DNA processes via its multiple phosphorylation sites. RPA-2 is phosphorylated on multiple N-terminal residues during the cell cycle by cyclin-dependent kinase 1 (CDK1)/ cyclin B [13] [14] and in response to DNA damage, when it is hyperphosphorylated by checkpoint kinases, including ATM (ataxia telangiectasia mutated), ATR (ATM and Rad3-related) and DNA-PK (DNA-dependent protein kinase) [15]. RPA-3 is thought to stabilize the RPA heterotrimer but very little is known about this protein [16].

Unlike other eukaryotes, little is known about RPA in trypanosomatids, protozoa that appear early in the evolution of eukaryotes. In 1992, Brown and collaborators purified the RPA complex from *Crithidia fasciculata* and demonstrated that RPA is a nuclear protein that can stimulate DNA polα but cannot replace human RPA in the SV40 DNA replication reaction [17]. In *Leishmania amazonensis*, LaRPA-1 has relevant structural differences compared with RPA-1 from humans and yeast [18]. In *Leishmania major*, LmRPA-1 accumulates in the DNA and co-localizes with an LmHus1 homologue after treatment with hydroxyurea (HU) and camptothecin, but the interaction of these two proteins has not been confirmed, so trypanosomatids RPA involvement in the repair/damage response remains obscure [19]. Despite this evidence, in *Leishmania*, only RPA-1 has been studied while RPA from *T*. *cruzi* and *T*. *brucei* have never been described. Here, we first cloned, expressed and purified RPA-1 and RPA-2 from *T*. *cruzi* (respectively TcRPA-1 and TcRPA-2) and showed that this protein complex is indeed a single stranded DNA binding protein that interacts with DNA through the TcRPA-1 subunit. Then, we suggest that TcRPA participates in canonical functions in DNA metabolism, such as replication and repair pathways. Finally, we were able to show that the reduction of TcRPA-2 expression by heterozygous knockout generation slowed down S-phase and consequently cell proliferation. Moreover, while cell growth was impaired in TcRPA-2 heterozygous knockout cells, a better efficiency in differentiation from epimastigote to metacyclic trypomastigote forms and metacyclic trypomastigote infection was observed, suggesting that the delay of cell cycle progression is linked to differentiation in *T*. *cruzi*.

## Methods

### Modeling and molecular dynamics simulations

A flexible linker containing 21 residues that connect OBF2 and OBF3 on TcRPA-1 and its 20 C-terminal residues do not show homology with any protein DNA-binding structure. Therefore, we created separated models for OBF1/OBF2 of TcRPA-1 (TcRPA-1-OBF12 model, corresponding to the 1–254 region) and for OBF3 of TcRPA-1 (TcRPA-1-OBF3 model, corresponding to the 272–445 region). Chain A of the crystal structure of DBD-A and DBD-B of RPA1 from *Homo sapiens* (PDB ID 1JMC; [20]) was selected as the best template for an initial *in silico* model for TcRPA-1-OBF12 (score: 342.7; E-value: 3e-57; identity: 41%). Chain C (Replication protein A 32 kDa subunit; RPA2) of the crystal structure of the human RPA trimerization core (PDB ID 1L1O; [21]) was selected for the initial *in silico* model of TcRPA-1-OBF3 (score 292.4; E-value: 1.9e-51; identity: 33%). Regarding TcRPA-2, the OB-fold domain corresponds to the 32–173 region of the protein, and chain B (Replication protein A 32 kDa subunit) of the crystal structure of the human RPA trimerization core (PDB ID 1L1O;[21]) was selected for the initial *in silico* model of this region (called herein as TcRPA-2-OBF model) (score 265.83; E-value: 2.9e-42; identity: 35%). The best templates for the TcRPA-1 and TcRPA-2 models were chosen according to data obtained from the profile-based threading method program Phyre2 [22]. Subsequently, these initial models were subjected to molecular dynamics (MD) simulations executed by GROMACS (Groningen Machine for Chemical Simulation) v.4.5.3 [23] in the presence of explicit water molecules. The protonation states of the charged groups were set according to pH 7.0. Counter ions were added to neutralize the system, and the Charmm27 force field [24] was chosen to perform the MD simulations. First, 200 ps of MD simulation with position restraints applied to the protein (PRMD) was executed to relax the system gently. Then, 100 and 50 ns of unrestrained MD simulations were performed, respectively to the TcRPA-1 and TcRPA-2 models, to evaluate the stability of the structures. The final *in silico* models of TcRPA-1-OBF12, TcRPA-1-OBF3 and TcRPA-2-OBF were deposited into the ModelArchive database [25] with the respective following access codes: ma-azgkp, ma-anz6r and ma-asqiv.

### Cell culture and differentiation

Exponentially growing *T*. *cruzi* epimastigotes (Y strain) were cultured in liver infusion tryptose (LIT) medium supplemented with 10% fetal bovine serum at 28°C [26]. The heterozygous knockout lineage (RPA2+/-) was cultured in the same conditions as above in the presence of 0.5 mg/ml of hygromycin B. In order to induce DNA damage, epimastigote cells were treated in PBS with 75μM of cisplatin for 1 h at 28°C. Cells were then centrifugated and maintained in culture refresh medium for 72 h. *T*. *cruzi* metacyclic trypomastigote forms were obtained using an *in vitro* differentiation assay as previously described [27]. Briefly, epimastigotes (5 x 10^8^) were harvested by centrifugation at 8,500 x *g* and incubated at 28°C for 2 h in 1 ml of artificial triatomine urine (TAU, 190 mM NaCl, 17 mM KCl, 2 mM CaCl_2_, 2 mM MgCl_2_, 8 mM phosphate buffer pH 6.0). Thereafter, the parasites were incubated in 25 cm^2^ culture flasks with 10 ml of TAU3AAG medium (TAU supplemented with 10 mM L-proline, 50 mM L-glutamate, 2 mM L-aspartate, 10 mM glucose) for 72 h. The metacyclic trypomastigotes were counted using a Neubauer chamber.

### Protein expression, purification and refolding and antibody production

**Fig 1:**
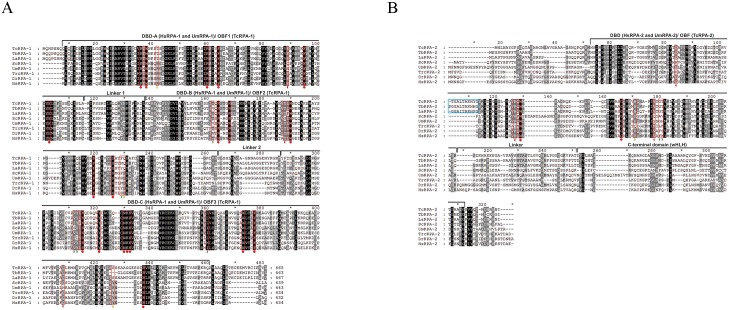
Amino acid sequence analysis of RPA-1 and RPA-2 from trypanosomatids and upper eukaryotes. (A) Amino acid sequence alignment of Replication Protein A1 from *Trypanosoma cruzi* (TcRPA-1; GI: 71664069), *Trypanosoma brucei* (TbRPA-1; GI: 788025025), *Leishmania amazonensis* (LaRPA-1; GI: 40317150), *Saccharomyces cerevisiae* (ScRPA-1, GI: 6319321), *Ustilago maydis* (UmRPA-1; GI: 428698228), *Tribolium castaneum* (TrcRPA-1, GI: 91094635), *Danio rerio* (DrRPA-1, GI: 167621451) and *Homo sapiens* (HsRPA-1; GI: 4506583). The red boxes highlight the residues involved in DNA binding on RPA1 crystal structures from *Homo sapiens* (PDB IDs 1JMC; 1L1O) and *Ustilago maydis* (PDB ID 4GNX). The OB-fold domains are called DNA binding domains (DBD, A-F) in hRPA. (B) Amino acid sequence alignment of RPA-2 from Trypanosoma cruzi (TcRPA-2; GI: 71410456), Trypanosoma brucei (TbRPA-2; GI: 788003719), *Leishmania amazonensis* (LaRPA-2; Contig APNT01001884.1, location 3085–4068 obtained at TritrypDB v. 9.0 Release 25), *Saccharomyces cerevisiae* (ScRPA-2, GI: 67230733), *Ustilago maydis* (UmRPA-2; GI: 428698227), *Tribolium castaneum* (TrcRPA-1, GI: 282400156), *Danio rerio* (DrRPA-1, GI: 836469293) and *Homo sapiens* (HsRPA-2; GI: 4506585). The red boxes highlight the residues involved in DNA binding on RPA2 crystal structures from *Homo sapiens* (PDB IDs 1L1O) and *Ustilago maydis* (PDB ID 4GNX). The symbols under the red boxes indicate conservation of these residues between the sequences: red circle for conservation in all sequences; red semi-circle for conservation in majority of eukaryotes including trypanosomatids; yellow semi-circle for conservation on eukaryotes but not in trypanossomatids; green semi-circle for non-conservation in the sequences. The blue box highlights the insertion of ten (*Leishmania amazonensis* and *Trypanosoma* cruzi) and eleven (*Trypanosoma brucei*) residues observed in RPA2 from trypanosomatids. The OB-fold domains are called DNA binding domains in hRPA.

To produce the recombinant proteins, TcRPA-1 and TcRPA-2 coding sequences (accession number: TcCLB.510901.60 and TcCLB.510821.50, respectively, http://tritrypdb.org/tritrypdb/) were amplified by PCR from *T*. *cruzi* genomic DNA and inserted into the pGEM-T easy vector (Promega). To produce separately RPA-1 OBF1, OBF2, and OBF3, specific primers were used to amplify each OBF accordingly to aligment of Fig 1 (OBF1: GCTAGCTGCAACACCCGAGC and AAGCTTTTACGCCAAAGAAATCTGACTCG; OBF2: GCTAGCAAGCAGCGGGAGGTG and AAGCTTTTAAGAGAGCGAAGAAACATCGC; OBF3: GCTAGCTATTTTGACGACATTTCCGC and AAGCTTTTACTGGCGCTTTTCCTCG). The amplified three fragments were inserted into pGEM-T easy vector (Promega). After, the TcRPA-1 and TcRPA-2 coding sequences were removed from pGEM-T easy and inserted into pET-28a(+), with a 6XHis-tag to facilitate protein purification, and transformed into *E*. *coli* Bl21(DE3). Protein expression was induced using 1 mM isopropyl thio-β-d-galactopyranoside (IPTG) at 37°C for an additional 3 h. The cells were harvested by centrifugation (3,200 x g, 10 min, 4°C) and suspended in lysis buffer (50 mM Tris-HCl pH 8.0, 50 mM NaCl, 10 mM EDTA pH 8.0 and 1X protease inhibitor cocktail (Roche)). Then, the cells were disrupted by sonication and incubated with 5U DNase I (Thermo Fischer Scientific), followed by centrifugation (18,000 x g, 10 min, 4°C). Both recombinant proteins were found in the insoluble fraction. The rTcRPA-1 that accumulated in inclusion bodies was solubilized in a buffer containing 20 mM glycine pH 10.0, 20 mM NaCl, 7 M urea and 1 mM β-mercaptoethanol. A pre-purification step before affinity chromatography was necessary to obtain rTcRPA-1 in a purified form [28]. To obtain purified rTcRPA-1, the bacterial extract was first purified by anion exchange chromatography on a HiTrap column of Q-Sepharose XL. Proteins were eluted with a linear gradient of elution buffer. The elution fraction was then subjected to affinity chromatography. The samples were loaded into a Niquel Sepharose column (Histrap HP- GE life Sciences) previously equilibrated with starting buffer (20 mM Tris-HCl pH 7.0, 0.5 M NaCl, 7 M urea, 71 μl/l β-mercaptoethanol). Recombinant proteins were eluted using a linear gradient of 25–500 mM imidazole in elution buffer (20 mM Tris-HCl pH 7.0, 0.5 M NaCl, 7 M urea and 500 mM imidazole). The purified recombinant proteins were subjected to dialysis in renaturing buffer (20 mM Tris-HCl pH 7.0 and 20 mM NaCl) at 4°C. Heparin (50 μg/ml) was added to each protein suspension before dialysis to prevent precipitation (see [28] for details). rTcRPA-1 was used to raise polyclonal antibodies as previously described [29]. rTcRPA-2 was submitted to customize specific antibodies (Proteimax, São Paulo).

### Mass spectrometry

Protein bands were excised from the SDS-polyacrylamide gel and subjected to in-gel trypsin digestion [30] and mass spectrometric analysis by LC-MS/MS. Peptide samples were automatically injected onto a trap column (5 cm, 100 μm I.D. x 360 μm O.D.) packed in-house with Jupiter C-18 10 μm resin (Phenomex, Torrence, CA, USA) in tandem with a C-18 analytical column (10 cm, 75 μm I.D. x 360 μm O.D.) packed in-house with Aqua 5 μm resin (Phenomex, Torrence, CA, USA). Peptides were eluted with a linear gradient of 5% to 40% buffer B (0.1% formic acid in acetonitrile) over 25 min at a flow rate of 200 nl/min controlled by a nano HPLC system Easy-nLC II (Thermo, San Jose, CA, USA). The eluate was electrosprayed on a LTQ Orbitrap Velos (Thermo, San Jose, CA, USA) by an electrospray nano-flow interface with 2.0 kV on the capillary. For the MS, the spectrometer was operated in a positive mode, and spectra were acquired in the m/z range of 200–2,000 with 60,000 resolution at 400 m/z using data dependent acquisition (DDA) where the top 10 most intense ions per scan were fragmented by collision-induced dissociation (CID). The minimal signal threshold to trigger a data-dependent scan was set to 5,000 cps. The repeat count for dynamic exclusion was set to 1, and the repeat duration was set to 30 s. The dynamic exclusion duration was set to 15 s and a list size of 500. MS data were analyzed using Mascot (version 2.4.1) against the UniProt database restricted to *Trypanosoma* (102,903 entries, downloaded on February 13^th^, 2014, with a peptide mass tolerance of 10 ppm and fragment mass tolerance of 0.5 Da). An iodoacetamide derivative of cysteine and the oxidation of methionine were specified in Mascot as fixed and variable modifications, respectively.

### Circular dichroism spectroscopy

CD measurements of rTcRPA-1 and rTcRPA-2 were obtained over the spectral ranges of 195–260 nm using a JASCO J-815 spectropolarimeter (JASCO Spectroscopic Co., Ltd., Japan) equipped with a Peltier thermo-controller. The experiments were performed at 293 K using an optical path length of 0.5 nm, a scanning speed of 100 nm/min, a response time of 1 s, a bandwidth of 2 nm and a data pitch of 0.5 nm. Twenty spectra were acquired, averaged and corrected for the buffer solution (baseline) in the presence and absence of single stranded DNA of 24 bp (ssDNA24) and then normalized to the residual molar ellipticity [θ]. The CD spectra of both buffer and ssDNA24 had negligible signals on the concentrations tested. rTcRPA-1 and rTcRPA-2 were analyzed in buffer containing 20 mM Tris-HCl and 20 mM NaCl, and the effect of single stranded DNA binding on CD spectra was evaluated by the addition of 150 pmol of single stranded DNA of 24 bp to the protein samples. Deconvolution of the CD spectra was performed using the Dichroweb online server [31] with the CDSSTR algorithm and reference set 4 [32].

### Electrophoretic mobility shift assay (EMSA)

Single-stranded DNA oligonucleotides were labeled with DIG-11-ddUTP by terminal transferase using a Dig Gel Shift kit (Roche). The gel shift assays were performed using 1 μg of TcRPA-1 or increased concentrations (1, 2 and 4 μg) of TcRPA-2 or of each truncated mutants, and fixed concentrations of 0.155 pmol of labeled oligonucleotide, 0.1 μg of poly-L-lysine, 1 μg poly[d(I-C)], 4 μl of 5X binding buffer (100 mM HEPES pH 6.0, 5 mM EDTA, 50 mM (NH_4_)_2_SO_4_, 5 mM DTT, Tween 20 1% (w/v), 150 mM KCl) in a final volume of 20 μl. The samples were maintained at room temperature for 15 min and then applied to a non-denaturing 6% gel (acrylamide/bis-acrylamide 37.5:1) run at 80 V in 0.25 X TBE buffer. Samples were then transferred onto a nylon membrane at 400 mA for 30 min in 0.25 X TBE and were fixed by UV light for 15 min. Detection of labeled oligonucleotides was performed using the Dig Gel Shift kit (Roche), according to the manufacturer's instructions.

### Immunofluorescence assays

Exponentially growing epimastigotes were pulsed with 100 μM of EdU (Click-iT Edu Image Kit, Invitrogen) for 5 minutes for replication assays or treated with 20 mM of hydroxyurea or UV radiation for DNA damage assays. The cells were pelleted, washed with PBS and fixed with 4% (v/v) paraformaldehyde in PBS for 20 min at room temperature. The cells were permeabilized with 0.1% Triton X 100 for 5 min and washed three times with PBS. After, the cells were incubated with anti-LmRPA-1 [19], anti-TcRPA-2 or anti-CPD (Cosmo Bio) primary rabbit antibodies for 1 h. Before incubation with anti-CPD, the cells were treated with 2 M HCl for 10 min at 37°C. The coverslips were rinsed three times with PBS and incubated with goat anti-rabbit IgG conjugated to Alexa Fluor 555 for 1 h. After washing the coverslips three times in PBS, the slides were mounted with VectaShield containing DAPI (Vector). For the EdU assay, after permeabilization, the cells were first incubated with a Click-iT reaction cocktail for 30 min at room temperature. Images were acquired through a z-series of 0.2 μm using a 100X 1.35NA lens and Cell R software in an Olympus IX81 microscope. Images were deconvolved using Autoquant X2.1.

### Immunoprecipitation (IP) and western blot analyses

*T*. *cruzi* epimastigote forms (1 x 10^7^ parasites/m1) in mid-log phase were harvested and processed to obtain a protein extract as previously described [33]. After, 100 μg of this protein extract was used as the input in IP assays, in conjunction with 10 μg of rabbit anti-TcRPA-2. The IP assays were performed using Dynabeads Protein A (Novex by Life Technologies), with a crosslinking step to avoid co-elution of the antibody heavy and light chains with TcRPA-1 or TcRPA-2, according to the manufacturer’s instructions. At the end of the assay, 50% of each IP eluate and 10% of the input were fractionated by 12% SDS-PAGE and transferred to nitrocellulose membranes. The membranes were probed with rabbit anti-TcRPA-1 or anti-TcRPA-2 as primary antibodies. A goat anti-rabbit IgG (H+L) HRP conjugate (Bio-Rad) was used as a secondary antibody. The reactions were revealed using the ECL western blotting analysis system (GE Healthcare).

### Generation of heterozygous RPA-2 knockout cells

Flanking sequences of the *TcRPA-2* gene were amplified from genomic DNA with a pair of primers: RPA2_KpnI + RPA2_SalI (5’ flank, 410 bp) and RPA2_BamHI + RPA2_XbaI (3’ flank, 389 bp). The TcRPA-2 flanking fragments were inserted into pTc2KO-hyg, which carries the hygromycin resistance gene (S1 Fig). The construction of this plasmid along with pTc2KO-neo (with G418 resistance gene) is described in the supplementary information. The 5’ flanking region was cloned between the KpnI and SalI sites whereas the 3’ flanking region was cloned between the BamHI and XbaI sites, resulting in the recombinant plasmid pTc2KO-RPA2-hyg. The targeting cassette was amplified from pTc2KO-RPA2-hyg with primers RPA2_KpnI and RPA2_XbaI and was used to transfect *T*. *cruzi* epimastigote forms as previously described [34]. Briefly, epimastigote forms at 2 x 10^7^/ml were pelleted, washed in PBS and resuspended in an electroporation solution (140 mM NaCl, 25 mM HEPES and 0,74 mM Na_2_HPO_4_ pH 7,5). Parasites were transferred into an electroporation cuvette (0.2 cm gap) (1.5 x 10^8^ cells in each one) followed by addition of 25 μg of the amplicon. Parasites transfected with no DNA were used as a control. After 10 min on ice, the samples were submitted to 2 pulses of 450 V, 500 μF in the Gene Pulser II Apparatus (Bio-Rad). Transfected parasites were cultured in LIT medium supplemented with hygromycin B (1 mg/ml) with passages every 8–10 days, until the death of the control parasites. In order to obtain clones of this lineage, cells were sorted by Flow Citometry and then one cell/well were maintained in LIT medium to start a clone culture. The correct insertion of the targeting cassette into the TcRPA-2 locus was confirmed by PCR from genomic DNA of RPA2 heterozygous knockout parasites using the following primer pairs: EXT5’_f + Hyg_r and EXT3’_r + Hyg_f. The primers EXT5’_f and EXT3’_r are flanking and outside of the site of the recombination cassette. Primer EXT5’_f is located 165 bp upstream of the cassette whereas primer EXT3’_r is located 139 bp downstream of the cassette.

### Infection assay

LLC-MK2 cells (Rhesus Monkey Kidney Epithelial Cells) (2x10^4^ cells/well) were cultured in a 6-well culture plate and maintained in DMEM supplemented with 10% FBS. After two hours, 10^6^ parasites, wild type or knockout, were added to each well. Twenty-four hours later, the medium was removed, and the cells were washed with PBS and fixed with 4% paraformaldehyde in PBS for 20 minutes, stained with eosin methylene blue for 30 minutes and dehydrated by acetone-xylene. The samples were mounted with Entellan and analyzed under a BX51 microscope (Olympus) to evaluate the number of infected cells in each group. A total of 100 cells were counted per replicate.

## Results

### *Trypanosoma cruzi* RPA (TcRPA) works as single stranded binding protein interacting with DNA through TcRPA-1

To confirm TcRPA as a single stranded binding protein in *T*. *cruzi*, we first analyzed amino acid sequence alignment between TcRPA-1 and TcRPA-2 from *T*. *cruzi* with RPA-1 and RPA-2 from other eukaryotes. An analysis of amino acid sequences of RPA-1 from *Saccharomyces cereviseae*, *Ustilago maydis*, *Tribolium castaneum*, *Danio rerio*, *Homo sapiens*, and the trypanosomatids *Leishmania amazonensis*, *Trypanosoma brucei*, and *T*. *cruzi* shows that, despite a few substitutions, the residues involved in ssDNA stacking in the OBF domains of *H*. *sapiens* ([20][21]) and *U*. *maydis* ([35]) crystal structures are conserved in the TcRPA-1 amino acid sequence (Fig 1A—red boxes). A peculiar feature that could be observed in this amino acid sequence alignment is the increase in the size of the linkers between the OBF domains of trypanosomatids and fungi compared with metazoan. Trypanosomatid RPA-1 sequences contain an insertion of two residues on a linker between OBF1 and OBF2 (linker 1). TcRPA-1, TbRPA-1, LaRPA-1, ScRPA-1 and UmRPA-1 contain an insertion of 7–8 residues between OBF2 and OBF3 (linker 2). The presence of these insertions can promote higher flexibility between OBF domains. Additionally, these insertions are rich in glycine and alanine residues, especially in linker 2 (Fig 1A). These data suggest a higher mobility between OBF domains in RPA-1 from trypanosomatids and fungi compared with metazoan RPA-1, which may have a more rigid tertiary structure. Finally, C-terminal region of RPA-1 trypanosomatids presents exclusive insertions that can contribute to the flexibility of this region on RPA-1 from these organisms. Regarding TcRPA-2, an analysis of amino acid sequence alignment containing RPA-2 from *H*. *sapiens*, *D*. *rerio*, *T*. *castaneum*, *Saccharomyces cerevisiae U*. *maydis*, *L*. *amazonensis*, *T*.*brucei* and *T*. *cruzi* shows that amino acids involved in DNA stacking on DBD-D of RPA-2 (according to the crystal structure of RPA-2 from *U*. *maydis* complexed to ssDNA ([35]) are also majorly conserved in trypanosomatids amino acid sequences (Fig 1B—red boxes). Similar to RPA-1, *T*. *cruzi* and *L*. *amazonensis* RPA-2 present the insertion of flexible residues also enriched in glycines and alanines (Fig 1B, blue box); however, this insertion was found not in the linkers but inside the OBF domain.

**Fig 2:**
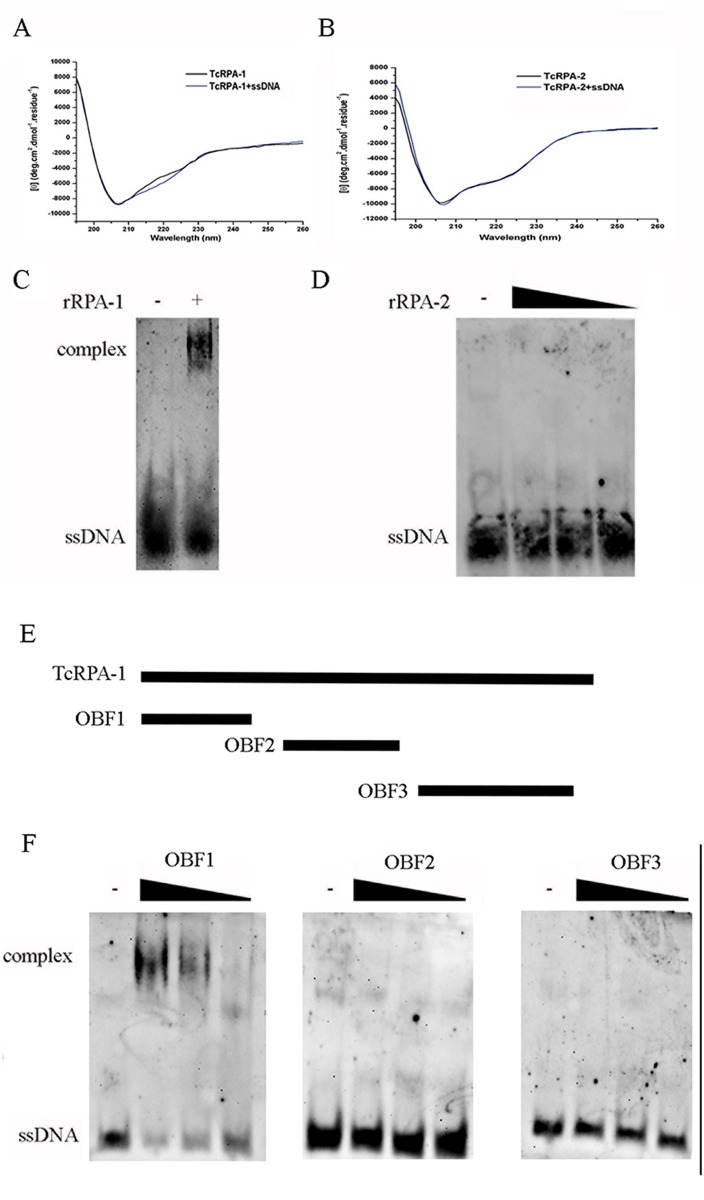
TcRPA-1 is able to bind ssDNA by OBF1. A and B. Circular dichroism (CD) spectra of (A) rTcRPA-1 and (B) rTcRPA-2 in the absence (black line) or presence of single stranded DNA of 24 bp (ssDNA24; blue line). A digoxin-labeled single stranded DNA (ssDNA) was maintained alone or in the presence (+) of recombinant TcRPA-1 (rRPA-1) (C) or of increasing concentrations of recombinant TcRPA-2 (rRPA-2) (D). The samples were analyzed by EMSA. E. Schematic representation of entire TcRPA-1 and three truncated mutants corresponding to each OBF domain. F. A digoxigenin-labeled ssDNA was maintained alone (-) or in the presence of recombinant OBF1, OBF2, and OBF3.

We then produced recombinants rTcRPA-1 and rTcRPA-2 (S2 and S3 Figs). The circular dichroism spectra of rTcRPA-1 and rTcRPA-2 showed a minimal number of values that varied from approximately 206–208 nm, indicating the presence of alpha-helices and a significant percentage of loops and disordered elements in both protein structures (Fig 2A and 2B). Similar CD results were obtained from full-length LaRPA-1 and truncated mutants of this protein ([28][18]). Deconvolution of the CD spectra showed 14% alpha-helices, 34% beta-sheets and 52% loops/disordered elements for rTcRPA-1 and 15% alpha-helices, 36% beta sheets and 49% loops/disordered elements for rTcRPA-2. These numbers are quite similar to those predicted for *in silico* models of TcRPA-1 and TcRPA-2, suggesting that these recombinant proteins assume the correct structure after refolding. To evaluate the impact of DNA binding on rTcRPA-1 and rTcRPA-2 secondary structures, CD spectra of both proteins were obtained in the presence of single-stranded DNA. The ssDNA increased the secondary structure of rTcRPA-1 because its CD spectra in the presence of ssDNA show an enhancement of the signal from 210 to 230 nm (Fig 2A and S4 Fig). Deconvolution of CD spectra of rTcRPA-1 with ssDNA24 shows a composition of 13% alpha-helices, 39% beta-sheets and 48% loops/disordered elements. In comparison with rTcRPA-1 spectra without ssDNA, the spectra in the presence of this DNA showed a decrease in disordered elements (52–48%) and an increase in beta-sheet secondary structure (34–39%). Regarding the CD spectra of rTcRPA-2, ssDNA did not cause any significative modification of the CD spectra of this protein because the CD spectra of rTcRPA-2 in the presence and absence of ssDNA24 were very similar (Fig 2B). These results suggested that only rTcRPA-1 is able to bind ssDNA. To confirm TcRPA-1-DNA interaction, rTcRPA-1 and rTcRPA-2 with ssDNA were analyzed by EMSA assay. Indeed, only rTcRPA-1 was able to form a complex with ssDNA (Fig 2C and 2D). It is important to note, however, that CD and EMSA assays (Fig 2) were performed using refolded recombinant proteins, which can contain structural variations compared with native ones.

To further assess the ability of RPA to bind single stranded DNA, we performed molecular modeling of TcRPA-1 and TcRPA-2 OBF domains. These models were performed first for OBF1/OBF2 of TcRPA-1 (TcRPA-1-OBF12 model, corresponding to the 1–254 region) and for OBF3 of TcRPA-1 (TcRPA-1-OBF3 model, corresponding to the 272–446 region) using Phyre2 software [22]. After, we performed molecular dynamics simulations (MD) of these initial models obtained by Phyre2. The final *in silico* models of TcRPA-1-OBF12 and TcRPA1-OBF3 were obtained after 100 ns of MD where 96.4% and 96.0%, respectively, of their residues were in favored and allowed regions of the Ramachandran plot [36]. Both models showed an overall good quality, as evaluated by ProSA-web [37] (Z-score = -6.02 and -5.24, respectively). The TcRPA-1-OBF12 *in silico* model has a similar tertiary structure compared to the DBD-A and DBD-B crystal structures from *H*. *sapiens* and *U*. *maydis*, especially for DBD-A (S5A and S5B Fig). Both crystal structures present an ssDNA binding channel that is extended in approximately a straight line from DBD-A to DBD-B [20][35]. This ssDNA binding channel is also observed in the TcRPA-1-OBF12 *in silico* model (S5C Fig), suggesting that ssDNA can bind to OBF1 and OBF2 of TcRPA-1 in a similar way in higher eukaryotes. To investigate this hypothesis, we have generated truncated RPA-1 containing just OBF1 or OBF2. However, we could observe only interaction of OBF1 with ssDNA by EMSA assay (Fig 2E and 2F). Regarding the TcRPA-1-OBF3 *in silico* model, the conserved amino acids involved in ssDNA binding are buried inside the protein, most likely blocking ssDNA access to the TcRPA-1 OBF3 binding site (S5D–S5G Fig). Therefore, TcRPA-1 does not seem to be able to bind DNA on OBF3, in contrast to higher eukaryotes. In fact, recombinant OBF3 was not able to interact with ssDNA in EMSA assay (Fig 2E and 2F). The crystal structure of the RPA complex with ssDNA of *U*. *maydis* showed that some regions of the linker between OBF2 and OBF3 of RPA1 is essential for the 8-to-30 nt DNA binding mode transition of RPA, which also includes RPA2 [35]. In this context, the 30-nt DNA binding mode seems to be unlikely to occur on trypanosomatids RPA, due to its inability to bind DNA from OBF3 domains on RPA1 and the unstable structure of the DNA binding site of RPA2 (see next section) of these organisms. Thus, the DNA binding mode of the RPA complex in trypanosomatids may involve fewer nucleotides and can be processed in a different way in relation to upper eukaryotes.

The Phyre2 software [22] predicted with 100% confidence a OBF domain in the 32–173 region and with 99.6% confidence as a winged-helix-loop-helix (wHLH) domain in the 205–252 region of TcRPA-2 (see Fig 1B) while we found that TcRPA-2 is not able to bind DNA. Therefore, we performed molecular modeling of the OBF domain of TcRPA-2 (TcRPA-2-OBF model, corresponding to the 32–173 region) using Phyre2. After, we performed molecular dynamics simulations (MD) of this initial model obtained by Phyre2. The final *in silico* model of TcRPA-2-OBF was obtained after 50 ns of MD where 99.3% of their residues are in favored and allowed regions of the Ramachandran plot [36]. The TcRPA-2-OBF model also demonstrated an overall good quality, as evaluated by ProSA-web [37] (Z-score = -5.03). The final *in silico* model of TcRPA-2-OBF adopts a very similar tertiary structure conformation compared to the crystal structures of RPA2 from *H*. *sapiens* and *U*. *maydis*, showing a similar DNA binding channel with these two proteins (S6A Fig). Amino acid sequence alignment shows that TcRPA-2 has a ten residue insertion rich in flexible residues (Fig 1B, blue box). Mapping the structural location of this insertion in the TcRPA-2-OBF *in silico* model shows that this insertion is located close to the DNA binding channel (S6B and S6C Fig). In fact, during molecular dynamics (MD) simulations, this region presented a high root mean square fluctuation (r.m.s.f.) of the main chain, adopting multiple positions during 50 ns of MD simulation, often even blocking the DNA binding channel (S6D Fig). These data suggest that the DNA binding site of trypanosomatid RPA-2 is more structurally unstable than upper eukaryotes, which can promote changes in the DNA binding affinity of RPA-2 from these organisms.

### TcRPA has canonical functions in DNA metabolism

**Fig 3:**
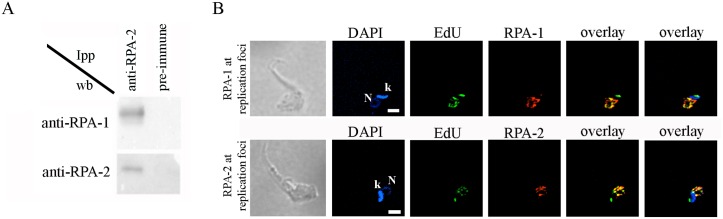
TcRPA-1 and TcRPA-2 form a complex *in vivo* and localize at replication foci. A. Cell extracts were immunoprecipitated with an anti-rTcRPA-2 antibody (anti-RPA2) or with pre-immune serum as a control. Samples eluted from the resin were submitted to SDS-PAGE, transferred onto nitrocellulose membranes and incubated with anti-rTcRPA-1 (anti-RPA-1) or anti-rTcRPA-2 (anti-RPA-2) antibodies. B. Cells were incubated with EdU, fixed, incubated with anti-rTcRPA-1 (anti-RPA-1) or anti-rTcRPA-2 (anti-RPA-2) and stained with DAPI. N-nucleus, k-kinetoplast.

**Fig 4:**
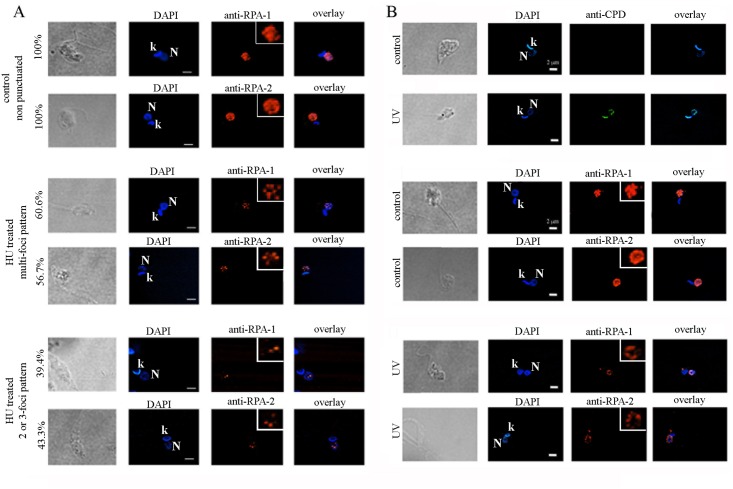
The pattern of TcRPA distribution changes after DNA damage. Cells were treated with HU (A) or irradiated with UV (B). Cells were fixed, permeabilized and incubated with anti-rTcRPA-1 (anti-RPA-1), anti-rTcRPA-2 (anti-RPA-2) and anti-CPD and stained with DAPI. In A, the percentage of cells found with each pattern of RPA distribution is indicated.

Because the DBD-C is involved in the RPA trimerization core in *H*. *sapiens* and *U*. *maydis* crystal structures, the structural differences observed on OBF-3 of TcRPA-1 raised questions about the formation of an RPA complex in *T*. *cruzi* [21] [35]. We performed immunoprecipitation with anti-TcRPA-2 and identified TcRPA-1 (Fig 3A), suggesting the presence of an RPA complex in these cells as found in other eukaryotes. As a single stranded binding protein complex, RPA affects DNA metabolism functions, such as DNA replication and DNA repair. To demonstrate these RPA functions in *T*. *cruzi*, we performed three distinct experiments. In the first experiment, cells were pulsed with the thymidine analogue EdU for a short period (5 min) to mark DNA replication factories, and the localization of TcRPA was evaluated. We found that RPA (detected with both anti-rTcRPA-1 and rTcRPA-2) co-localized with replication sites in nuclei, but not in kinetoplast (Fig 3B), most likely stabilizing single stranded DNA during nuclear DNA duplication. Next, we induced replicative stress by HU treatment and analyzed TcRPA-1 and TcRPA-2 nuclear localization by immunofluorescence assays. After HU treatment, the cells were synchronized at the G1/S transition [38] due to inhibition of DNA replication by the depletion of dNTP pools. We found that 40% of cells presented TcRPA-1 and TcRPA-2 in a 2-3-foci pattern and 60% of cells presented TcRPA-1 and TcRPA-2 in a multi-foci pattern contrasting with a dispersed nuclear localization observed in control cells (Fig 4A). Finally, we induced DNA damage through UV irradiation, and immunofluorescence with anti-CPD was used to demonstrate the presence of pyrimidine dimers induced by UV (Fig 4B). TcRPA-1 and TcRPA-2 were found in a punctuated pattern constrained at the nuclear periphery in UV irradiated cells while control cells presented TcRPA dispersed throughout the nuclear space (Fig 4B). In fact, in eukaryotes RPA localization at intra-nuclear foci is observed in response to DNA damage/replication stress, co-localizing with several recombination repair and checkpoint proteins [39] [40] [41]. Therefore, our data strongly suggest the involvement of TcRPA in DNA damage repair. Taken together, these data suggest that RPA has canonical functions in DNA metabolism in *T*. *cruzi*.

### Cell proliferation is impaired in TcRPA-2 heterozygous knockout parasites

**Fig 5:**
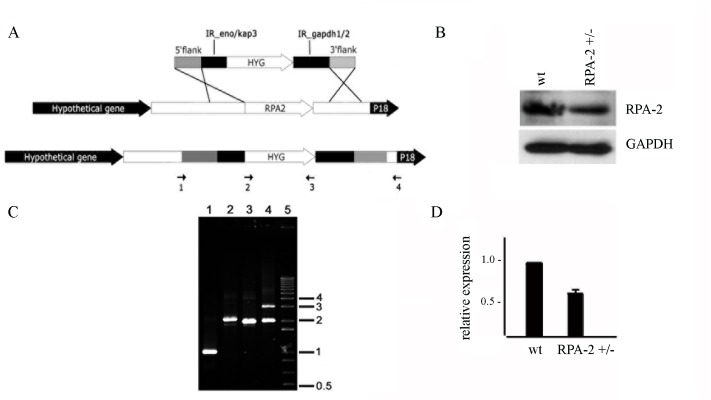
Generation of heterozygous knockout cells expressing reduced levels of TcRPA-2. A. Schematic representation of the TcRPA-2 locus (central panel) or the locus generated after replacement of the RPA-2 gene by gene targeting (bottom panel) with the recombination cassette carrying the hygromycin resistance gene (top panel). Arrows indicate the regions where the primers used in the experiments presented in B anneal. B. DNA extracted from RPA-2 heterozygous knockout cells was amplified using the primers presented in A. 1- Hyg_f (2) + Hyg_r (3) (1.026 bp); 2- Hyg_f (2) + EXT5’_r (4) (2.113 bp); 3- EXT5’_f (1) + Hyg_r (3) (1.994 bp); 4- EXT5’_f (1) + EXT3’_r (4). 5–1 kb plus DNA ladder marker (Invitrogen). The primers EXT5’_f and EXT3’_r anneal in regions outside of the recombination site. Thus, the two bands observed in lane 4 correspond to the amplified regions from the wild-type (2.070 bp) and targeted RPA-2 alleles (3.081 bp), which are present in the RPA-2+/- parasites. C. Cell extracts from control and RPA-2+/- cells were subjected to SDS-PAGE, transferred onto nitrocellulose membranes and incubated with anti-rTcRPA-2 or anti-GAPDH, which was used as a loading control. D. The intensity of the bands obtained with anti-rTcRPA-2 presented on C were normalized using the intensity of the GAPDH bands. Graph shows average and standard deviation of three independents experiments.

To perturb cell proliferation by DNA metabolic impairment, TcRPA-2 levels were reduced by heterozygous knockout cell generation. The replacement of an RPA-2 allele by insertion of the hygromycin selectable marker was confirmed using specific primers located inside or outside of the recombination cassette (Fig 5A and 5B). Using an anti-rTcRPA-2 antibody, we demonstrated that heterozygous knockout reduced the level of TcRPA-2 expression to 66.4% ± 3.2 (Fig 5C and 5D). We have also added efforts trying to generate RPA-2 null mutants. However, this mutant was not viable, evidencing that RPA is a fundamental protein for parasite survival.

**Fig 6:**
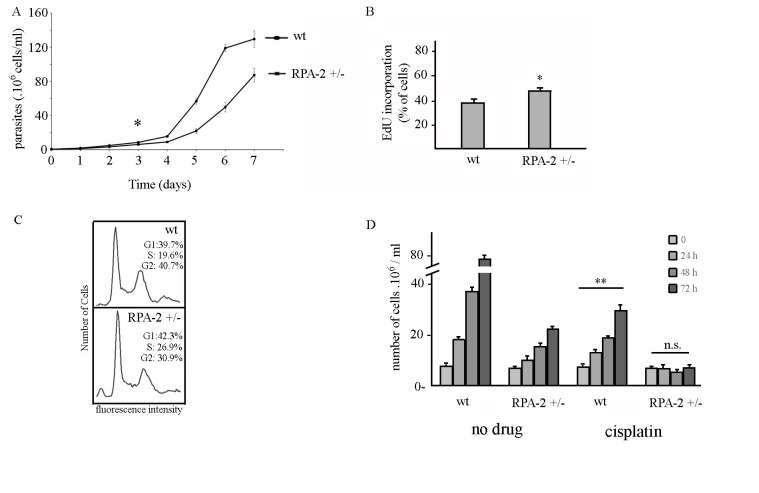
RPA-2 heterozygous knockout cells present growth and DNA replication impairment and S-phase progression delay. A. Growth curve of RPA-2+/- and wild type (wt) cells. * indicates the day during the growth curve that samples were taken to perform assays presented in B and C. B. Control and RPA-2+/- cells were maintained in the presence of EdU for 5 minutes and the percentage of labeled cells was determined. Graph shows average and standard deviation of three independents experiments. C. FACS analysis of wild type (wt) and RPA-2+/- cells labeled with propidium iodide. D. Growth culture of wild type (wt) and RPA-2+/- cells after treatment with 75μM of cisplatin or with no treatment (no drug). Statistical analyses were performed using a Student t-test, ** means p < 0.001; n.s. means p > 0.8.

The reduction of TcRPA-2 expression impairs cell growth (Fig 6A). Additionally, the percentage of cells labeled by EdU incorporation is higher in RPA-2 heterozygous knockout cells (Fig 6B), suggesting that S phase is longer when the TcRPA-2 level is reduced. The increment of S phase duration was confirmed by cell cycle analysis (Fig 6C). These data strongly suggest that lacking the TcRPA-2 subunit of the RPA complex slows down DNA replication, strengthening the role of TcRPA in this process. Moreover, a reduction of the cell growth of TcRPA-2 heterozygous knockout cells might be at least in part a consequence of a delay in DNA duplication.

### TcRPA2 heterozygous knockout parasites were not able to growth after genotoxic treatment

We took advantage of the generation of *TcRPA2 heterozygous knockout parasites* to better investigate the involvement of RPA in DNA damage response in *T*. *cruzi*. We used cisplatin to generate monoadducts as well as intra- and inter-strand crosslinks in DNA ([42]). After treatment, we followed the growth culture of wild type and TcRPA2 heterozygous knockout cells. In fact, TcRPA2 heterozygous knockout were not able to recover cell growth even after 72 h of treatment, while growth of wild type cells was observed 24 h after treatment (Fig 6D). This result adds evidence to the hypothesis suggested above that RPA is indeed an important player in DNA damage response.

### Differentiation and infection capacity is increased in TcRPA-2 heterozygous knockout cells

**Fig 7:**
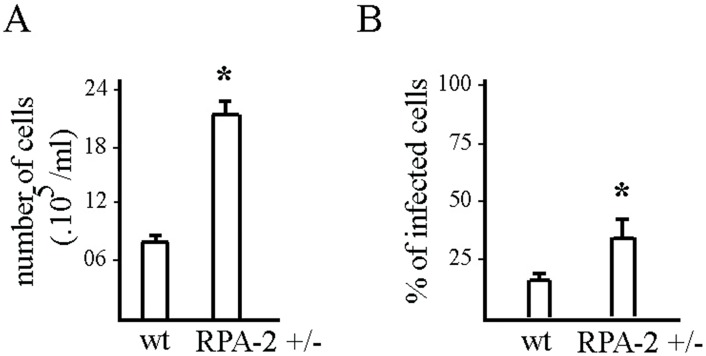
RPA-2+/- cells present a higher capacity of differentiation and infection compared with wild type cells. (A) The same amount of wild type (wt) and heterozygous knockout RPA-2+/- cells were submitted to metacyclogenesis and obtained metacyclic trypomastigotes were quantified. B. The same amount of wild type (wt) and heterozygous knockout RPA-2+/- metacyclic trypomastigotes were used to infect cells. After 24 h, the percentage of infected cells was determined. Statistical analyses were performed using a Student t-test, * means p < 0.01.

Finally, taking into account that our data show that the reduction in TcRPA-2 level impairs *T*. *cruzi* proliferation, we assessed whether TcRPA-2 heterozygous knockout also induced changes in *T*. *cruzi* differentiation. First, TcRPA-2 heterozygous knockout and wild type epimastigote forms were submitted to the *in vitro* metacyclogenesis process. Unlike the effect on *T*. *cruzi* proliferation, the reduction of the levels of TcRPA-2 in the heterozygous knockout parasites increased *T*. *cruzi* differentiation. In fact, three times more metacyclic trypomastigotes were obtained in heterozygous knockout cells when compared to wild type (Fig 7A). We also generated five different clones and analyzed the level of expression of RPA-2. All clones expressed the same amount of protein, and three analyzed also demonstrated quite similar growth and ability to differentiate onto metacyclic (S7 Fig). These data evidence that hemi-knockout lineage RPA-2+/- is homogenous. In addition, the percentage of LLC-MK2 cells infected by metacyclic trypomastigote forms from heterozygous knockout parasites (37%) was higher than that from wild type parasites (21%) (Fig 7B). Taken together these data showed that the reduction of TcRPA-2 expression increases the capacity of epimastigote differentiation and metacyclic trypomastigote infection.

## Discussion

In this manuscript, we performed *in silico*, *in vitro* and *in vivo* analysis of *T*. *cruzi* RPA-1 and RPA-2 subunits. We could observe, by alignment analysis, that both TcRPA-1 and TcRPA-2 indeed present OB-fold domains, including residues that are important for RPA-DNA interaction in mammalian and fungi RPA [20][35]. However, *in vitro* analysis demonstrated that TcRPA-1 can interact with single stranded DNA while TcRPA-2 cannot, although we can not exclude the possibility that the RPA-2 tridimensional structure obtained after refolding of insoluble protein could be the cause of lacking of RPA-2-ssDNA interaction. According to our molecular model of the predicted tridimensional structure of TcRPA-1, OBF1 and OBF2 are the major OB-folds responsible for RPA-1-DNA interactions, forming an open binding channel between these two OB-fold domains. *In vitro* assay showed interaction only between ssDNA and OBF1. It is important to note, however, that OBF2 may need OBF1 to increase its affinity for ssDNA as occurs in mammalian cells ([8]). In contrast, OBF3 of TcRPA-1 seems to be unable to interact with DNA because the residues involved in DNA binding are buried within the protein. These results differ from the crystallographic structure of hRPA-1, where the residues of DBD-C are exposed to solvent and hRPA can interact with DNA via multiple binding modes, which can include DBD-C and also DBD-D (present in RPA-2) in some binding modes [8]. Our molecular model of TcRPA-2 suggests that this protein does not have the capacity to interact with DNA due to an exclusive insertion, present in trypanosomatids RPA-2 inside OBF/DBD-D, which is unstable and adopts multiple positions that very often occlude the DNA binding channel during MD simulations. In conclusion, we suggest that *T*. *cruzi* RPA interacts with DNA only through TcRPA-1. Regarding the third subunit of RPA, we performed a thorough and low stringent *in silico* search in the local files of annotated proteins and we have found a *bona fide* candidate for *T*. *cruzi* RPA-3 (TcCLB.507011.150). However, further investigation is necessary in order to demonstrate that it is indeed part of RPA complex.

Alignment of TcRPA-1 with fungi and mammalian sequences showed that TcRPA-1 lacks a 70-N domain (DBD-F), important for interaction with proteins involved in DNA damage and checkpoint responses [12]. The lack of a 70-N domain appears to be a common characteristic of trypanosomatids RPA-1 because it has been previously observed in *Leishmania* [18] and *Chrithidia* [17]. Despite its absence, we demonstrated here that the pattern of TcRPA localization changes in response to DNA damage induced by UV irradiation. Moreover, TcRPA localization at the nuclear periphery after UV irradiation matches with DNA damage localization at the nuclear periphery observed using an anti-CPD antibody. In addition, the constriction of repair machinery, using LmHus1 as a marker, at the nuclear periphery after genotoxic treatment was previously shown in *Leishmania* [19]. Moreover, treatment of TcRPA-2 heterozygous knockout parasites with genotoxic compound showed that RPA is indeed involved in DNA damage response, even lacking the 70-N domain.

We also demonstrated that TcRPA is involved in DNA replication and the replicative stress response, with canonical functions previously demonstrated in other organisms [6][43][15] [21][44]. In accordance with its involvement in DNA replication, the reduction of TcRPA-2 expression levels via heterozygous knockout cells slowed down S-phase progression and cell growth. TcRPA is also involved in metacyclogenesis in *T*. *cruzi* because heterozygous knockout epimastigote cells presented a 3.0-fold increased ability to differentiate onto metacyclic trypomastigotes. As cited above, the effect of RPA reduction on differentiation was demonstrated before in *Drosophila* neuroepithelial differentiation into neuroblasts, where the authors suggest that a delay of cell cycle progression is causally linked with differentiation [5]. Other non-canonical functions of RPA were previously described in *Leishmania braziliensis*, where RPA-1 is able to bind the untranslated region of *HSP70* mRNA, suggesting that RPA from trypanosomatids can also be an RNA-binding protein [45]. Moreover, *in silico* analysis presented here showed that TcRPA-1 has more flexible linkers that connect OB-fold domains compared to yeast and mammalian RPAs. Because it was recently suggested that the size and flexibility of these linkers are directly involved in RPA function [46], it is possible that trypanosomatids RPA is indeed involved in additional responses, such as the ones discussed above.

Further investigations are necessary to understand how TcRPA could be involved in the differentiation process and also to analyze whether TcRPA participates directly in the metacyclogenesis process or whether the delay of cell cycle progression triggered by TcRPA-2 reduction increases *T*. *cruzi* differentiation. It is important to note, however, that there is no obligatory coupling between cellular decisions to proliferate and to differentiate, even though the two behaviors are often controlled at the same time [47]. Because metacyclogenesis in *T*. *cruzi* is triggered by a low pH, a shift in temperature and nutrient restriction, it has been proposed that the altered redox state could be a metabolic integrator in cells under multiple stress conditions, triggering or regulating the differentiation process [1]. Therefore, we cannot exclude the possibility that RPA could be one of the molecules participating in the process of identify alteration in the redox state. In fact, the alteration of the redox state can regulate human RPA activity [48].

Our knowledge of the molecules involved in the detection of environmental changes and transduction of these changes triggering *T cruzi* differentiation is limited. For this reason, increasing the knowledge in the field of the *T*. *cruzi* differentiation process, including RPA in this scenario, will be important to provide valuable information regarding new therapeutic targets.

## Supporting Information

S1 Fig Diagrams of the plasmids pTc2KO-hyg (A) and pTc2KO-neo (B) used for gene replacement in *T*. *cruzi*. NeoR = neomycin resistance gene, HygR = hygromycin resistance gene, IR_eno/kap3 = the intergenic region between the genes encoding for enolase (gene ID: TcCLB.511529.90) and kap3 (gene ID: TcCLB.511529.80) from *T*. *cruzi*. IR_gapdh1/2 = the intergenic region between the two copies of the *T*. *cruzi* GAPDH genes (gene ID: TcCLB.506943.50 and TcCLB.506943.60). To construct the pTc2KO-hyg, the plasmid pTc2KO-neo was digested with *Hind*III and *Eco*RI to remove the *neo* gene and ligated with the hygromycin-resistance (hyg) gene.
(TIFF)

S2 Fig Expression, purification and antibodies generated against rTcRPA-1 and rTcRPA-2. rTcRPA-1 (A) and rTcRPA-2 (D) were expressed in the presence of IPTG in a prokaryotic system. C. Expressed rTcRPA-1 was purified first by anion exchange and then using a Niquel column. D. rTcRPA-2 was purified using a Niquel column. C and F. Protein extracts from *T*. *cruzi* cells were submitted to SDS-PAGE and transferred onto nitrocellulose membranes that were incubated with anti-rTcRPA-1 (C), anti-rTcRPA-2 (F) or normal serum as a negative control. (G) RPA-1 mutants corresponding to OBF1, OBF2, and OBF3 were expressed and purified using a Niquel column. Eluted proteins were analyzed by SDS-PAGE.
(TIF)

S3 Fig Recombinant proteins are in fact TcRPA-1 and TcRPA-2. Obtained purified recombinant proteins (S1 Fig) were subjected to gel trypsin digestion followed by mass spectrometric analysis using LC-MS/MS. (*) means significant peptides with p<0,05 and (**) means unique peptides.
(TIF)

S4 Fig TcRPA-1 changes its folding after interaction with ssDNA. Circular dichroism (CD) spectra of rTcRPA-1 in the absence (black line) or presence of single stranded DNA of 24 bp (ssDNA24; blue line). Each graph was obtained from an independent experiment.
(TIF)

S5 Fig Molecular modeling of OB-fold domains 1 and 2 (TcRPA-1-OBF12 model) and OB-fold domain 3 (TcRPA-1-OBF3 model) of Replication Protein Factor A1 from *Trypanosoma cruzi*. (A) Cartoon representation of C_α_ superposition between the crystal structures of DNA binding domains A and B (DBD-A and DBD-B) from *Ustilago maydis* (yellow; PDB ID 4GNX; [35]) and *Homo sapiens* (green; PDB ID 1JMC;[20]) and TcRPA-1-OBF12 *in silico* model (wheat). (B) Root mean square deviation (r.m.s.d.) of TcRPA-1-OBF12 *in silico* model along 100 ns of molecular dynamics (MD) simulation calculated by GROMACS. (C) Cartoon representation of TcRPA-1-OBF12 *in silico* model highlighting 138–146 and 211–215 loops (in magenta) and the residues involved in ssDNA binding of RPA1 crystal structures that are conserved in TcRPA-1 (blue sticks). In TcRPA-1, similarly to *H*. *sapiens* and *U*. *maydis* DBD-A/DBD-B crystal structures, the ssDNA has free access to both OB-fold domains. (D) Cartoon representation of the crystal structure of DNA binding domain C (DBD-C) in the presence of single-stranded DNA (ssDNA; in orange) from *Ustilago maydis* (PDB ID 4GNX;[35]). Residues involved in ssDNA binding are highlighted in blue sticks. (E) Cartoon representation of the crystal structure of DNA binding domain C (DBD-C) from *Homo sapiens* (PDB ID 1L1O; [21]. (F) Cartoon representation of TcRPA-1-OBF3 *in silico* model. Residues involved in ssDNA binding of the crystal structure of DBD-C from *U*. *maydis* are highlighted in blue sticks. (G) Root mean square deviation (r.m.s.d.) of TcRPA-1-OBF3 *in silico* model along 100 ns of molecular dynamics (MD) simulation calculated by GROMACS. Whereas in *U*. *maydis* and *H*. *sapiens* crystal structures there is a DNA binding channel with residues involved in DNA binding exposed to solvent, in the TcRPA-1-OBF3 *in silico* model, this channel is occluded and the residues that could be involved in DNA binding are buried within the protein.
(TIFF)

S6 Fig Molecular modeling of the OB-fold domain of Replication Protein Factor A2 from *Trypanosoma cruzi* (TcRPA-2-OBF model). (A) Cartoon representation of C_α_ superposition between crystal structures of the OB-fold domain from RPA2 from *U*. *maydis* (purple; PDB ID 4GNX;[35]) and from RPA2 from *H*. *sapiens* (yellow; PDB ID 1L1O;[21]) and the *in silico* model of the OB-fold domain of RPA2 from *T*. *cruzi* (green; TcRPA-2-OBF model). The ssDNA presented in the *U*. *maydis* RPA2 crystal structure is also displayed in the cartoon (in orange and blue). (B) Cartoon representation of the final TcRPA-2-OBF *in silico* model. The residues of TcRPA-2-OBF that aligns with RPA2 from *U*. *maydis* and the same position of residues involved in DNA binding (see Fig 2) in this crystal structure (PDB ID 4GNX) are highlighted in sticks. The 85–95 region that presents several structural positions during MD simulations is highlighted in red. (C) Cartoon representation of C_α_ superposition of the crystal structure of the OB-fold domain from RPA2 from *U*. *maydis* (purple; PDB ID 4GNX) and the final TcRPA-2-OBF *in silico* model (green). The residues (except glycines) involved in DNA binding in the crystal structure of RPA2 from *U*. *maydis* are highlighted in sticks. The residues of TcRPA-2-OBF (except glycines) that align with RPA2 from *U*. *maydis* in the same position of the residues involved in DNA stacking (see Fig 2) in this crystal structure (PDB ID 4GNX) are highlighted in sticks. (D) Root mean square fluctuation (r.m.s.f.) of the final TcRPA-2-OBF *in silico* model. It is possible to observe that the 85–95 region presents a high r.m.s.f. during MD simulations and, consequently, adopts several structural positions along 50 ns of MD simulations. (E) Root mean square deviation (r.m.s.d.) of the TcRPA-2 *in silico* model from the MD simulations. R.m.s.f. and r.m.s.d of MD simulations were calculated using GROMACS.
(TIFF)

S7 Fig Different clones of RPA-2 heterozygous knockout cells express quite similar levels of RPA-2 and present similar growth and differentiation capacity. A. Top panel indicates cell extracts from RPA-2+/- total culture and three different clones of RPA-2+/- cells were subjected to SDS-PAGE, transferred onto nitrocellulose membranes and incubated with anti-rTcRPA-2 or anti-GAPDH, which was used as a loading control. Bottom panel indicates quantification of RPA-2 expression. Experiment was done in duplicate. All clones showed non-significative (n.s.) differences when compared to RPA-2+/- using Student’s t-test. B. Growth curve of wilt type (wt), RPA-2+/- total culture, and three different clones obtained from RPA-2+/- (cl1, cl2, and cl3). C. The same amount of wild type (wt), heterozygous knockout RPA-2+/- cells, and three different clones obtained from RPA-2+/- (cl1, cl2, and cl3) were submitted to metacyclogenesis and obtained metacyclic trypomastigotes were quantified.
(TIF)
