# Supplementary figures and images for: Replication Protein A Presents Canonical Functions and Is Also Involved in the Differentiation Capacity of Trypanosoma cruzi
Source: PLoS Negl Trop Dis. 2016 Dec 16;10(12):e0005181. doi: 10.1371/journal.pntd.0005181 (PMC5161316; doi:10.1371/journal.pntd.0005181)

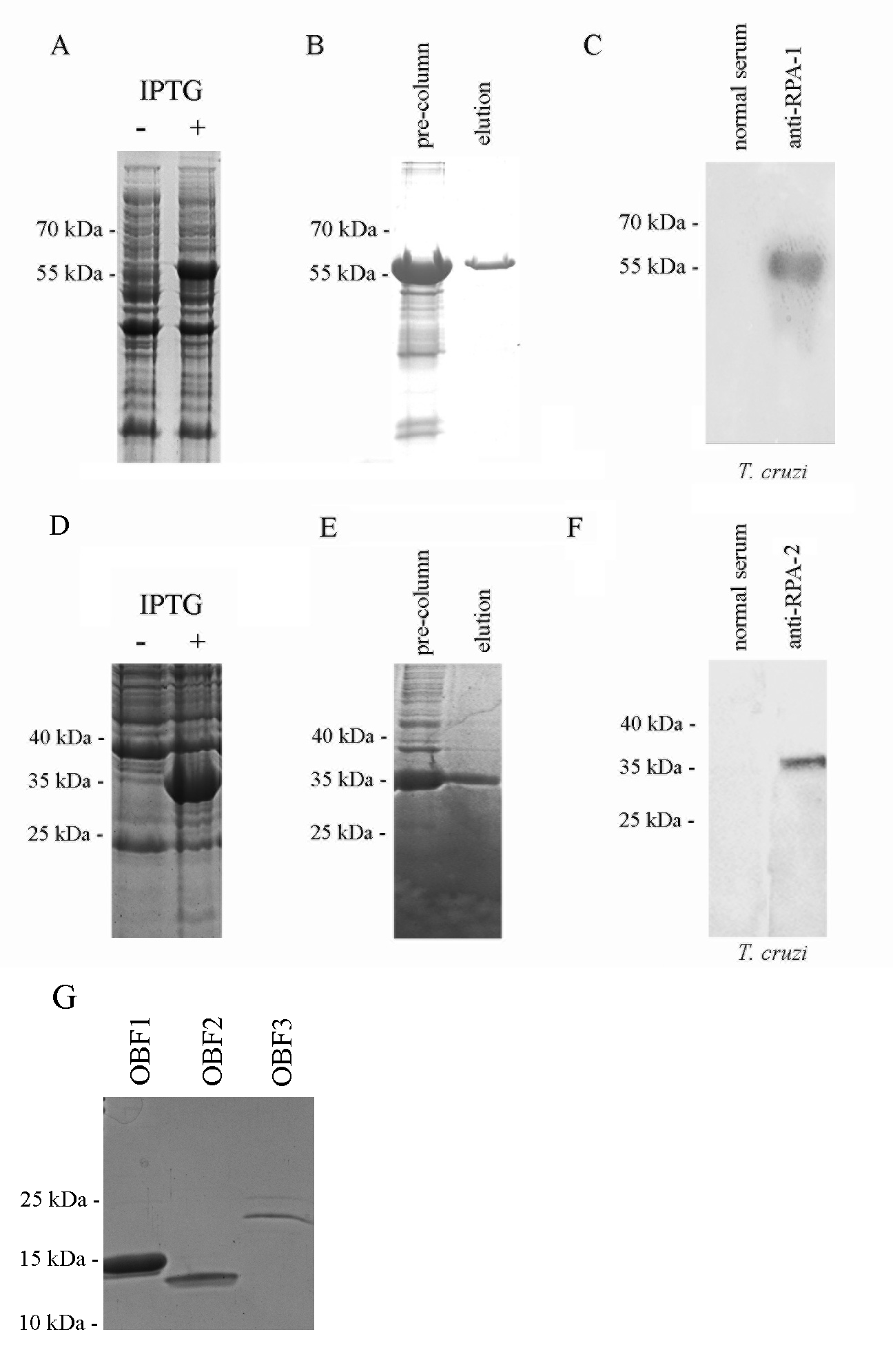

Supplement: S2 Fig — rTcRPA-1 (A) and rTcRPA-2 (D) were expressed in the presence of IPTG in a prokaryotic system. C. Expressed rTcRPA-1 was purified first by anion exchange and then using a Niquel column. D. rTcRPA-2 was purified using a Niquel column. C and F. Protein extracts from T. cruzi cells were submitted to SDS-PAGE and transferred onto nitrocellulose membranes that were incubated with anti-rTcRPA-1 (C), anti-rTcRPA-2 (F) or normal serum as a negative control. (G) RPA-1 mutants corresponding to OBF1, OBF2, and OBF3 were expressed and purified using a Niquel column. Eluted proteins were analyzed by SDS-PAGE. (TIF) [file pntd.0005181.s002.tif]

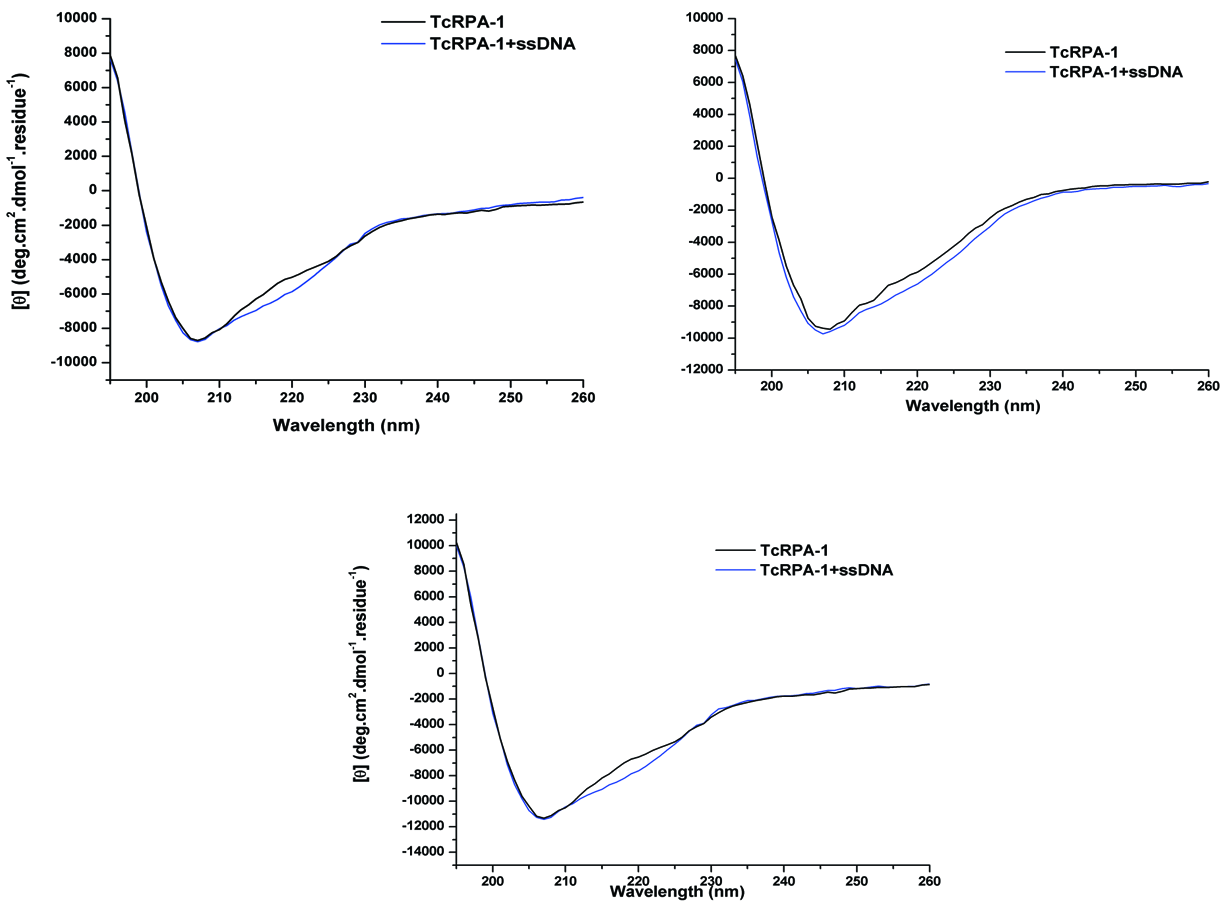

Supplement: S4 Fig — Circular dichroism (CD) spectra of rTcRPA-1 in the absence (black line) or presence of single stranded DNA of 24 bp (ssDNA24; blue line). Each graph was obtained from an independent experiment. (TIF) [file pntd.0005181.s004.tif]

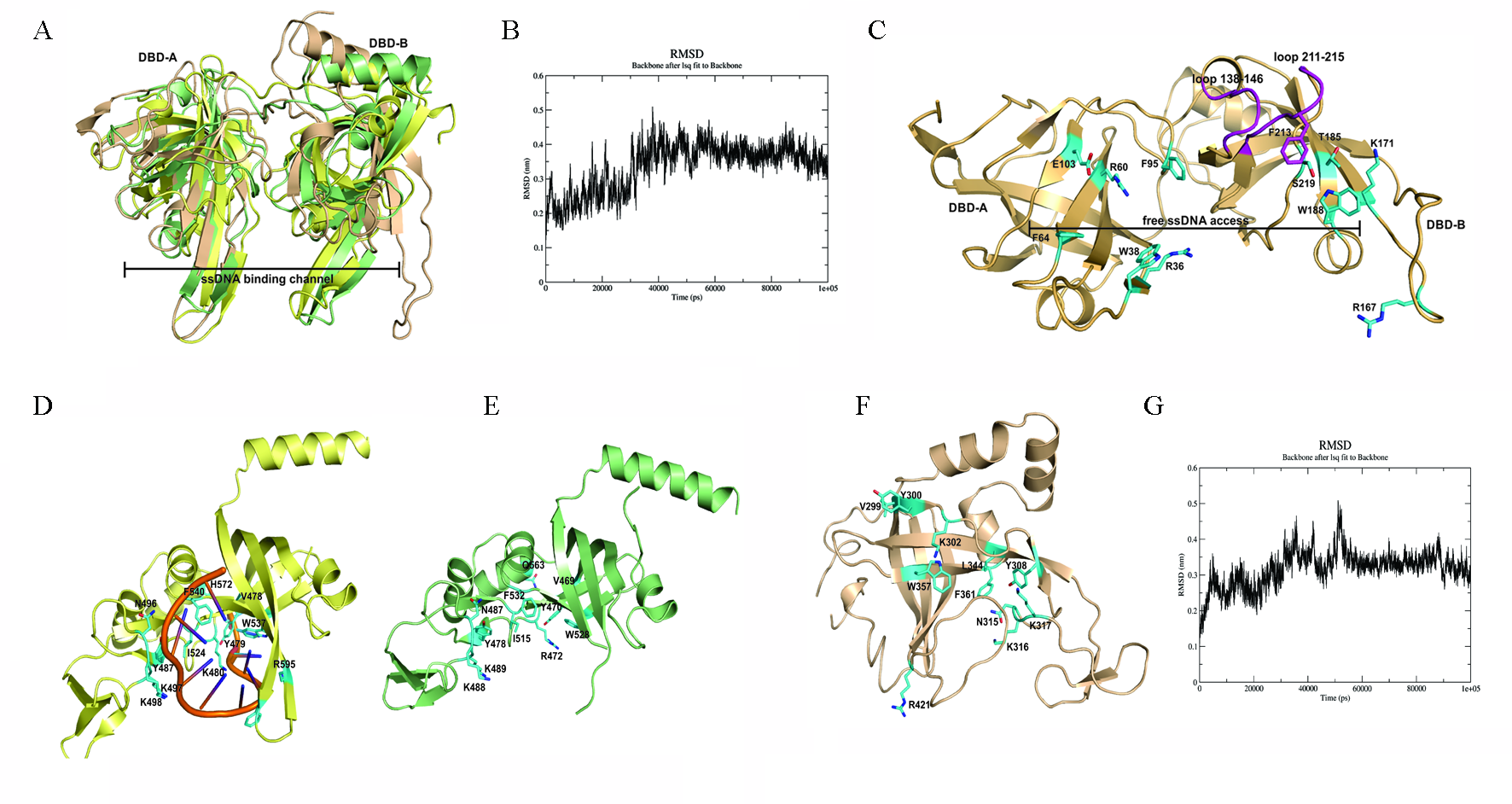

Supplement: S5 Fig — (A) Cartoon representation of Cα superposition between the crystal structures of DNA binding domains A and B (DBD-A and DBD-B) from Ustilago maydis (yellow; PDB ID 4GNX; [35]) and Homo sapiens (green; PDB ID 1JMC;[20]) and TcRPA-1-OBF12 in silico model (wheat). (B) Root mean square deviation (r.m.s.d.) of TcRPA-1-OBF12 in silico model along 100 ns of molecular dynamics (MD) simulation calculated by GROMACS. (C) Cartoon representation of TcRPA-1-OBF12 in silico model highlighting 138–146 and 211–215 loops (in magenta) and the residues involved in ssDNA binding of RPA1 crystal structures that are conserved in TcRPA-1 (blue sticks). In TcRPA-1, similarly to H. sapiens and U. maydis DBD-A/DBD-B crystal structures, the ssDNA has free access to both OB-fold domains. (D) Cartoon representation of the crystal structure of DNA binding domain C (DBD-C) in the presence of single-stranded DNA (ssDNA; in orange) from Ustilago maydis (PDB ID 4GNX;[35]). Residues involved in ssDNA binding are highlighted in blue sticks. (E) Cartoon representation of the crystal structure of DNA binding domain C (DBD-C) from Homo sapiens (PDB ID 1L1O; [21]. (F) Cartoon representation of TcRPA-1-OBF3 in silico model. Residues involved in ssDNA binding of the crystal structure of DBD-C from U. maydis are highlighted in blue sticks. (G) Root mean square deviation (r.m.s.d.) of TcRPA-1-OBF3 in silico model along 100 ns of molecular dynamics (MD) simulation calculated by GROMACS. Whereas in U. maydis and H. sapiens crystal structures there is a DNA binding channel with residues involved in DNA binding exposed to solvent, in the TcRPA-1-OBF3 in silico model, this channel is occluded and the residues that could be involved in DNA binding are buried within the protein. (TIFF) [file pntd.0005181.s005.tiff]

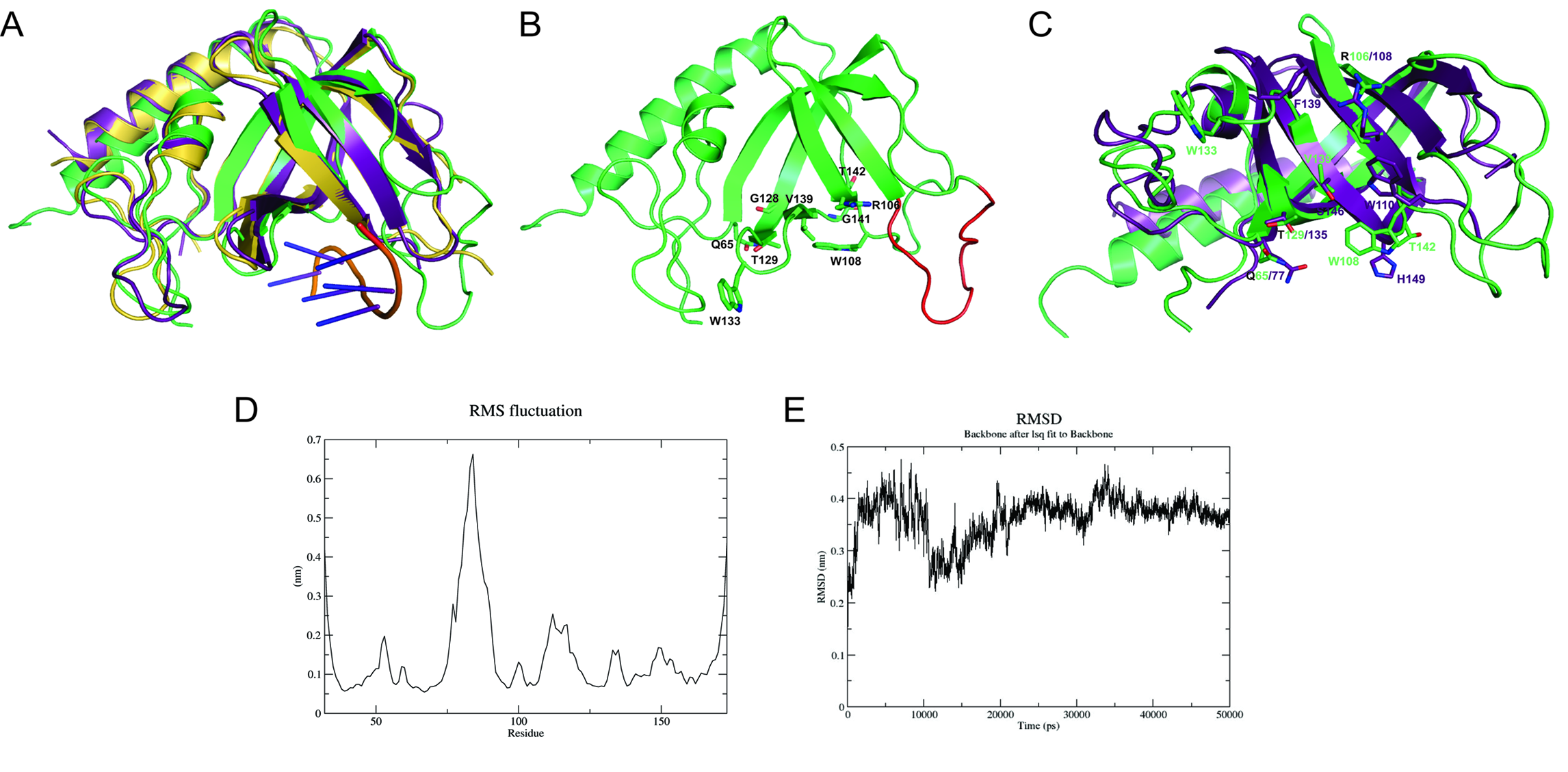

Supplement: S6 Fig — (A) Cartoon representation of Cα superposition between crystal structures of the OB-fold domain from RPA2 from U. maydis (purple; PDB ID 4GNX;[35]) and from RPA2 from H. sapiens (yellow; PDB ID 1L1O;[21]) and the in silico model of the OB-fold domain of RPA2 from T. cruzi (green; TcRPA-2-OBF model). The ssDNA presented in the U. maydis RPA2 crystal structure is also displayed in the cartoon (in orange and blue). (B) Cartoon representation of the final TcRPA-2-OBF in silico model. The residues of TcRPA-2-OBF that aligns with RPA2 from U. maydis and the same position of residues involved in DNA binding (see Fig 2) in this crystal structure (PDB ID 4GNX) are highlighted in sticks. The 85–95 region that presents several structural positions during MD simulations is highlighted in red. (C) Cartoon representation of Cα superposition of the crystal structure of the OB-fold domain from RPA2 from U. maydis (purple; PDB ID 4GNX) and the final TcRPA-2-OBF in silico model (green). The residues (except glycines) involved in DNA binding in the crystal structure of RPA2 from U. maydis are highlighted in sticks. The residues of TcRPA-2-OBF (except glycines) that align with RPA2 from U. maydis in the same position of the residues involved in DNA stacking (see Fig 2) in this crystal structure (PDB ID 4GNX) are highlighted in sticks. (D) Root mean square fluctuation (r.m.s.f.) of the final TcRPA-2-OBF in silico model. It is possible to observe that the 85–95 region presents a high r.m.s.f. during MD simulations and, consequently, adopts several structural positions along 50 ns of MD simulations. (E) Root mean square deviation (r.m.s.d.) of the TcRPA-2 in silico model from the MD simulations. R.m.s.f. and r.m.s.d of MD simulations were calculated using GROMACS. (TIFF) [file pntd.0005181.s006.tiff]

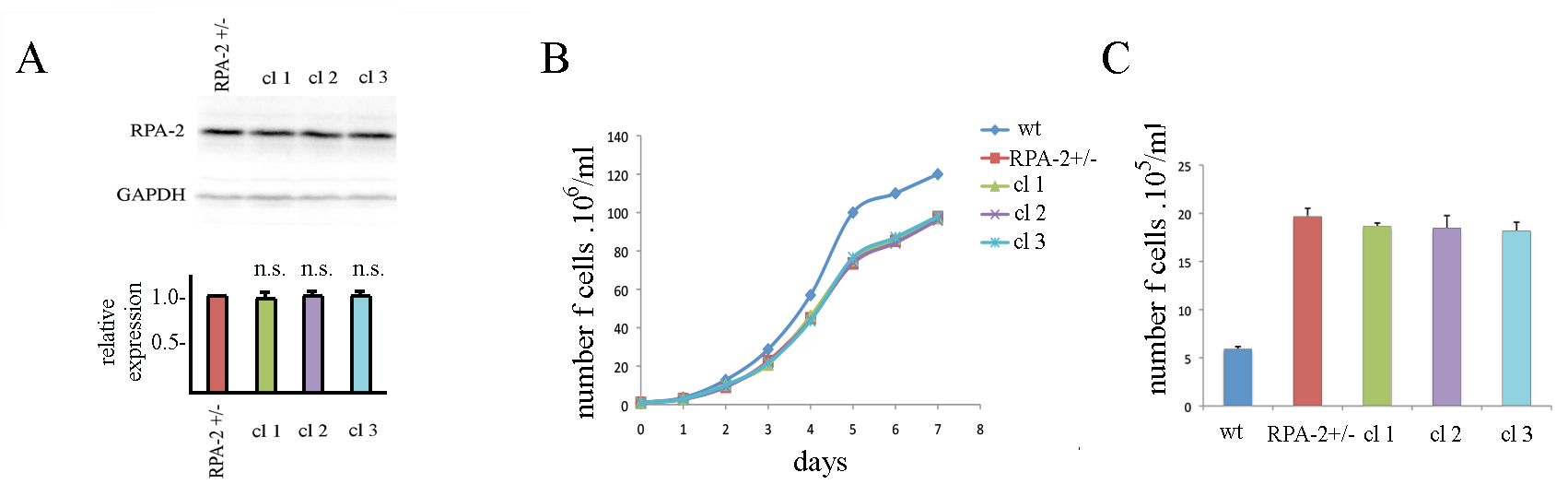

Supplement: S7 Fig — A. Top panel indicates cell extracts from RPA-2+/- total culture and three different clones of RPA-2+/- cells were subjected to SDS-PAGE, transferred onto nitrocellulose membranes and incubated with anti-rTcRPA-2 or anti-GAPDH, which was used as a loading control. Bottom panel indicates quantification of RPA-2 expression. Experiment was done in duplicate. All clones showed non-significative (n.s.) differences when compared to RPA-2+/- using Student’s t-test. B. Growth curve of wilt type (wt), RPA-2+/- total culture, and three different clones obtained from RPA-2+/- (cl1, cl2, and cl3). C. The same amount of wild type (wt), heterozygous knockout RPA-2+/- cells, and three different clones obtained from RPA-2+/- (cl1, cl2, and cl3) were submitted to metacyclogenesis and obtained metacyclic trypomastigotes were quantified. (TIF) [file pntd.0005181.s007.tif]
